# Positive Autoregulation Delays the Expression Phase of Mammalian Clock Gene *Per2*

**DOI:** 10.1371/journal.pone.0018663

**Published:** 2011-04-14

**Authors:** Yukino Ogawa, Nobuya Koike, Gen Kurosawa, Tomoyoshi Soga, Masaru Tomita, Hajime Tei

**Affiliations:** 1 Institute for Advanced Biosciences, Keio University, Tsuruoka, Yamagata, Japan; 2 Systems Biology Program, Graduate School of Media and Governance, Keio University, Fujisawa, Kanagawa, Japan; 3 Mitsubishikagaku Institute of Life Science, Machida, Tokyo, Japan; 4 Department of Neuroscience, University of Texas Southwestern Medical Center, Dallas, Texas, United States of America; 5 Theoretical Biology Laboratory, RIKEN Advanced Science Institute, Wako, Saitama, Japan; 6 Graduate School of Natural Science and Technology, Kanazawa University, Kanazawa, Ishikawa, Japan; Pennsylvania State University, United States of America

## Abstract

In mammals, cellular circadian rhythms are generated by a transcriptional-translational autoregulatory network that consists of clock genes that encode transcriptional regulators. Of these clock genes, *Period1* (*Per1*) and *Period2* (*Per2*) are essential for sustainable circadian rhythmicity and photic entrainment. Intriguingly, *Per1* and *Per2* mRNAs exhibit circadian oscillations with a 4-hour phase difference, but they are similarly transactivated by CLOCK-BMAL1. In this study, we investigated the mechanism underlying the phase difference between *Per1* and *Per2* through a combination of mathematical simulations and molecular experiments. Mathematical analyses of a model for the mammalian circadian oscillator demonstrated that the slow synthesis and fast degradation of mRNA tend to advance the oscillation phase of mRNA expression. However, the phase difference between *Per1* and *Per2* was not reproduced by the model, which implemented a 1.1-fold difference in degradation rates and a 3-fold difference in CLOCK-BMAL1 mediated inductions of *Per1* and *Per2* as estimated in cultured mammalian cells. Thus, we hypothesized the existence of a novel transcriptional activation of *Per2* by PER1/2 such that the *Per2* oscillation phase was delayed. Indeed, only the *Per2* promoter, but not *Per1*, was strongly induced by both PER1 and PER2 in the presence of CLOCK-BMAL1 in a luciferase reporter assay. Moreover, a 3-hour advance was observed in the transcriptional oscillation of the *delta-Per2* reporter gene lacking cis-elements required for the induction by PER1/2. These results indicate that the *Per2* positive feedback regulation is a significant factor responsible for generating the phase difference between *Per1* and *Per2* gene expression.

## Introduction

The circadian clock controls daily rhythms of biological activities such as the sleep/wake pattern in many organisms. The cellular mechanism of the mammalian clock has been characterized as a transcriptional-translational autoregulatory network that consists of clock genes encoding transcriptional regulators [1]. In this network, the circadian expressions of clock genes peak one after another, and their expression phases may determine the timing of internal events such as metabolism [2]. Both the *Per1* and *Per2* genes are rhythmically transactivated by the CLOCK-BMAL1 heterodimer, which binds to the E/E′-box motifs in their promoter regions [3]–[6], as well as *Cryptochrome1* (*Cry1*) [7], [8] and *Rev-erbα* [9], [10], which are components of negative feedback loops in the mammalian circadian clock. The transcriptional activation of *Per1* and *Per2* by CLOCK-BMAL1 is repressed by CRY1 and CRY2 [5]–[8], whereas REV-ERB*α* represses *Bmal1* transcription via the transcription factor binding site RORE [9], [10]. These negative feedback regulations guarantee sustainable circadian oscillations.

The *Per1* and *Per2* genes are essential to sustain the circadian rhythm, and the behaviors of *Per1^−/−^*/*Per2^−/−^* double mutant mice are arrhythmic [11], [12]. Intriguingly, the oscillation phase of *Per2* mRNA lags behind that of *Per1* by approximately 4 hours in the suprachiasmatic nucleus (SCN), which is the master circadian regulator in the brain, and other peripheral tissues [13]–[17], though the oscillatory expressions of both *Per1* and *Per2* are assumed to be evoked by CLOCK-BMAL1 transactivity. The functions of PER1 and PER2 proteins (PER1/2) are partially redundant because both *Per1* and *Per2* single mutant mice are rhythmic under both light-dark (LD) and constant dark conditions [11], [12], [18]–[20]. However, the differing roles of PER1 and PER2 have also been documented in the different behaviors of *Per1^Brdm1^* and *Per2^Brdm1^* single mutant mice, which show abnormal responses to photic stimuli under light-dark conditions [20]. In this study, we investigated the mechanisms underlying the phase difference between *Per1* and *Per2* expression by a combination of mathematical simulations and molecular experiments. The elucidation of the regulatory mechanism of *Per1* and *Per2* expression should provide important clues about the robust self-sustainable oscillation and photic entrainment of the circadian clock.

Because the circadian regulatory network is a self-sustainable oscillatory circuit, it is of interest not only for cellular biology but also for computational biology. Thus, many mathematical models have been developed through the accumulation of biological knowledge [21]. Mathematical approaches enable us to test whether our current knowledge about the regulation of *Per1* and *Per2* expression is sufficient for explaining the phase difference of *Per1* and *Per2*. If the current knowledge is not sufficient, studies that incorporate mathematical models can yield predicted mechanisms that regulate gene expression to generate the phase difference, and these predictions can then be tested experimentally. By combining mathematical and experimental approaches, we report here that a new transcriptional regulation mechanism is needed to explain the phase difference in the expression of *Per1* and *Per2* mRNAs.

## Results

### *In silico* analysis of an mRNA expression phase in a current circadian oscillatory network model

**Figure 1:**
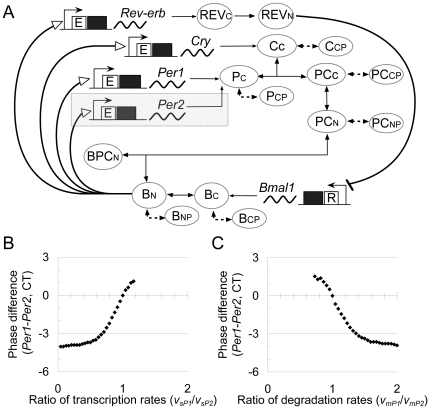
Effects of mRNA transcription and degradation rates on the *Per* mRNA expression phase. (A) Schematic representation of the circadian oscillatory network model used to compare the expression phases of *Per1* and *Per2* mRNAs, which was based on the Leloup and Goldbeter model [22]. The *Per2* gene transcription and translation are additionally introduced in the shaded region. A square, wave line, and circle indicate a gene, mRNA, and protein, respectively. Details are described in Text S1. (B, C) Variation in the phase difference between the *Per1* and *Per2* mRNA oscillations with varied (B) the proportion of the *Per1* transcription rate coefficient to the *Per2* transcription rate coefficient (***v_sP1_***/***v_sP2_***) and (C) the proportion of the *Per1* degradation rate coefficient to the *Per2* degradation rate coefficient (***v_mP1_***/***v_mP2_***). The rate coefficient of transcription and degradation of Per2 were fixed to 2.4 nM/h and 2.2 nM/h, respectively. The phase difference of *Per1* from *Per2* is indicated in circadian time (CT).

To analyze the mechanism that generates the oscillation phase difference between *Per1* and *Per2*, we employed a mathematical model of the circadian clock that included *Per*, *Cry*, *Bmal1*, and *Rev-erbα*, as proposed by Leloup and Goldbeter with following modifications [22]. We introduced the *Per1* and *Per2* genes instead of *Per* to compare their oscillation phases because *Per1* and *Per2* were not distinguished and the *Per* gene represented both of *Per1* and *Per2* in the original model (**Figure 1A**). The kinetics equations and parameters of *Per1* and *Per2* were the same as those of original *Per* except for the translation rate coefficient, which was divided in half because the PER protein represented the sum of the translational products of both genes. All kinetic parameters and reaction rate equations for the 20 variables are indicated in Table S1 (Model1) and Text S1.

As long as the transcriptional regulation of *Per1* and *Per2* is the same as hypothesized in the model, the observed phase difference between *Per1* and *Per2* mRNA oscillations is not likely to occur. However, possible difference in synthesis and/or degradation rates may cause the phase difference between *Per1* and *Per2*. We computationally estimated the dependency of oscillation phases on the transcription rate by varying the proportion of the *Per1* transcription rate coefficient (***v_sP1_***) to the *Per2* transcription rate coefficient (***v_sP2_***; **Figure 1B**). Similarly, the proportion of the *Per1* degradation rate coefficient (***v_mP1_***) to the *Per2* degradation rate coefficient (***v_mP2_***) was varied (**Figure 1C**). As shown in **Figure 1B and C**, slow synthesis or fast degradation of mRNA advanced the phase of oscillation. Indeed, the 4-hour phase lag of *Per2* mRNA behind *Per1* mRNA could be reproduced when the *Per1* transcription was 0.8-fold lower than that of *Per2* or when the *Per1* mRNA degradation was 2-fold more than that of *Per2*. If the transcription of *Per1* was much faster than that of *Per2* (i.e., ***v_sP1_***/***v_sP2_***≥1.2 in **Figure 1B**) or the degradation of *Per1* was much slower than that of *Per2* (i.e., ***v_mP1_***/***v_mP2_***≤0.7 in **Figure 1C**), oscillations did not occur, and the orbit converged to the steady state. From the numerical results, we conjectured that the transcriptional activity of *Per2* is higher than that of *Per1* or that the rate of *Per1* mRNA degradation is faster than that of *Per2*, which causes the observed phase difference between *Per1* and *Per2* mRNA oscillations.

In addition to the model proposed by Leloup and Goldbeter, other computational models for mammalian circadian clock, which reproduce the time-series data of clock gene mRNA and protein expression, have been also proposed [23], [24]. One of these models developed by Forger and Peskin including different kinetic parameters of *Per1* and *Per2* transcription did not reproduce the expression phase difference between *Per1* and *Per2*. Another model developed by Mirsky *et al.* reproduced the phase difference between *Per1* and *Per2* mRNA. Actually, the phase difference was generated by the different kinetic rates such as Hill coefficient and Michaelis constant of *Per1* and *Per2* transcription. However, the kinetic rates assumed in this model were not measured experimentally. Therefore tested the hypothesis that difference in kinetic rates between *Per1* and *Per2* dynamics can account for the phase difference by using the experimentally measured parameters.

### Synthesis and degradation rates of *Per1* and *Per2* mRNAs *in vitro*

**Figure 2:**
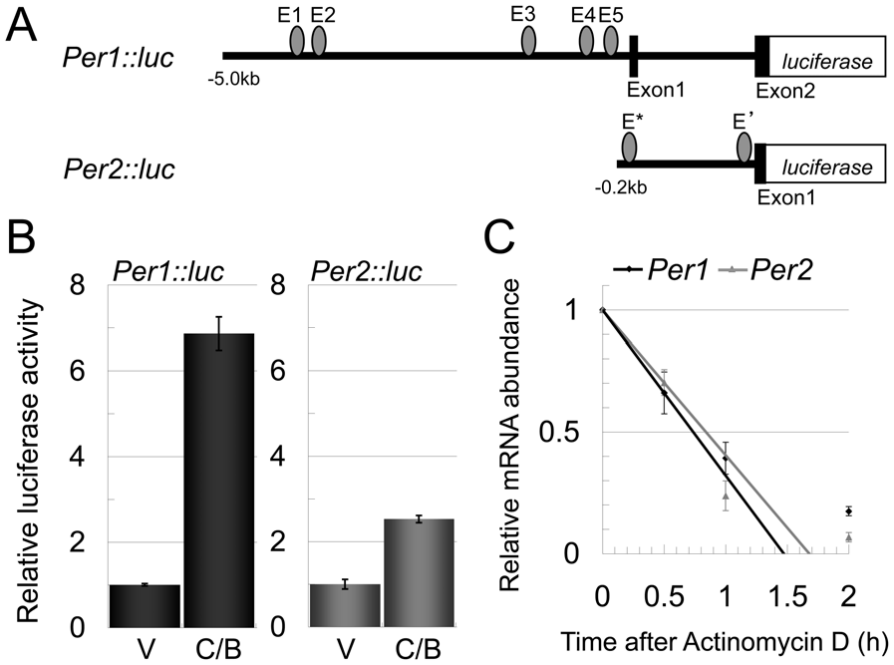
Quantitation and simulation of transcription intensities and degradation velocities of *Per1* and *Per2* mRNA. (A) Schematic representation of the *Per1::luc* and *Per2::luc* reporters. The *Per1* promoter driving the luciferase reporter (*Per1::luc*) contains the 6.7-kb region upstream of the translation-initiation codon and includes five E-boxes (CACGTG), and the *Per2* promoter driving the luciferase reporter (*Per2::luc*) contains 0.2-kb upstream of the first exon and includes two E-box like elements, E′ (CACGTT) and E* (CAGGTG). Filled boxes represent exons, and ellipses are E-boxes and E-box like elements. (B) Promoter activities of *Per1::luc* and *Per2::luc*. *Per1::luc* was activated 6.86±0.38 times and *Per2::luc* was activated 2.52±0.08 times by co-expression of CLOCK-BMAL1 with respect to their basal promoter activities, respectively. V indicates vector control and C/B indicates CLOCK and BMAL1 co-expression. Error bars indicate SEM determined from independent experiments in triplicate. (C) Initial velocities of *Per1* and *Per2* mRNA degradation in rat SCN-derived cultured cells. Cellular abundances of *Per1* and *Per2* mRNA were measured after actinomycin D treatment. The degradation slope of *Per1* mRNA was −0.68 and the mRNA half-life was 44.1 min, whereas the degradation slope of *Per2* mRNA was −0.60 and the mRNA half-life was 50.0 min. Error bars indicate SEM determined from independent experiments in quadruplicate, with the exception of the experiment for *Per1* 1 hour after treatment, which was performed in duplicate. See materials and methods for a detailed description of the experimental procedure.

To evaluate our mathematical estimation, we next measured the promoter activities of *Per1* and *Per2* as well as the degradation rates of these mRNAs *in vitro*. The promoter activities of *Per1* and *Per2* were measured by using two reporter genes *Per1::luc* and *Per2::luc*, in which the *Per1* [25] and *Per2* [5] promoters, respectively, were fused to the luciferase gene (**Figure 2A**). Both reporter genes were induced by *Clock* and *Bmal1* co-transfection; however, a 3-fold higher induction was observed in cells transfected with *Per1::luc* compared to *Per2::luc* (**Figure 2B**). The higher transcriptional activity of *Per1* did not produce the 4-hour phase advance in *Per1* expression compared to *Per2* because the increase in promoter activity should have delayed the oscillation phase as estimated by the previous mathematical analysis (**Figure 1B**). Subsequently, we examined the degradation rates of *Per1* and *Per2* mRNA in a cell line derived from the rat SCN (**Figure 2C**) [26]. Although the faster degradation of *Per1* satisfies a requirement for the advanced *Per1* oscillation phase compared to *Per2* in this model, neither the 1.1-fold faster rate of *Per1* degradation nor the 0.9-fold slower rate of *Per2* degradation estimated *in vitro* reproduced the 4-hour phase difference (**Figure 1C**).

**Figure 3:**
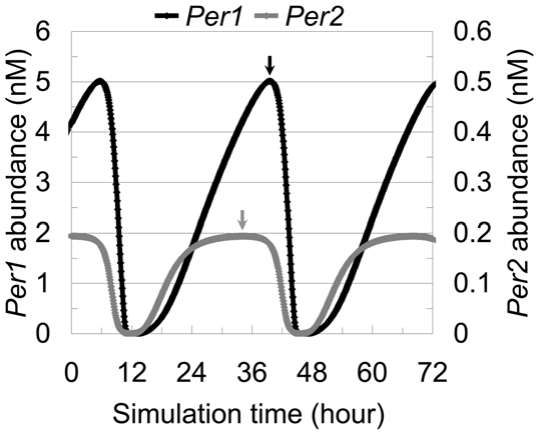
Measured synthesis and degradation rates do not reproduce the phase relationship between *Per1* and *Per2*. A simulation result of *Per1* and *Per2* mRNA expressions calculated by the model schematized in **Figure 1A** with the ratio of both transcription and degradation rates measured experimentally and applied as parameters. This model simulated the circadian oscillations in 33.8-hour period. After 1000 hours simulation, the first peak of *Per1* mRNA was set to simulation time 6. The *Per2* expression level was almost 25 times lower than that of *Per1* even though its corresponding transcriptional rate was just one-third of the original value, and the *Per2* phase was advanced by 5.4 hours, which was inconsistent with the experimentally observed results. Arrows indicate expression peaks.

Then, the combined effect of the transcription and degradation rate ratios on the phase difference was examined using our mathematical model. However, the oscillation phase of *Per2*, but not of *Per1*, was advanced by +5.4 hours (**Figure 3**). The differences observed in the promoter activities induced by CLOCK-BMAL1 and mRNA degradation rates could not reproduce the 4-hour phase difference between *Per1* and *Per2*.

### A new model including an additional feedback regulation to reproduce the phase delay of *Per2*

As described above, our modified model (Text S1, Eqs. **S1–S20**) with measured parameters could not reproduce the phase difference between *Per1* and *Per2* mRNA oscillations. Therefore, we hypothesized several models, including an additional transcriptional regulation that may account for the phase difference. The basic idea underlying our modeling was that a feedback regulation of *Per1* or *Per2* transcription by PER1/2 could be the basis for the observed phase difference between *Per1* and *Per2*. To express this idea, we studied i) positive feedback regulation of *Per2* transcription, ii) negative feedback regulation of *Per1* transcription, iii) positive feedback regulation of *Per1* transcription, and iv) negative feedback regulation of *Per2* transcription by PER1/2. We examined whether any of these mechanisms could potentially explain the observed phase difference.

**Figure 4:**
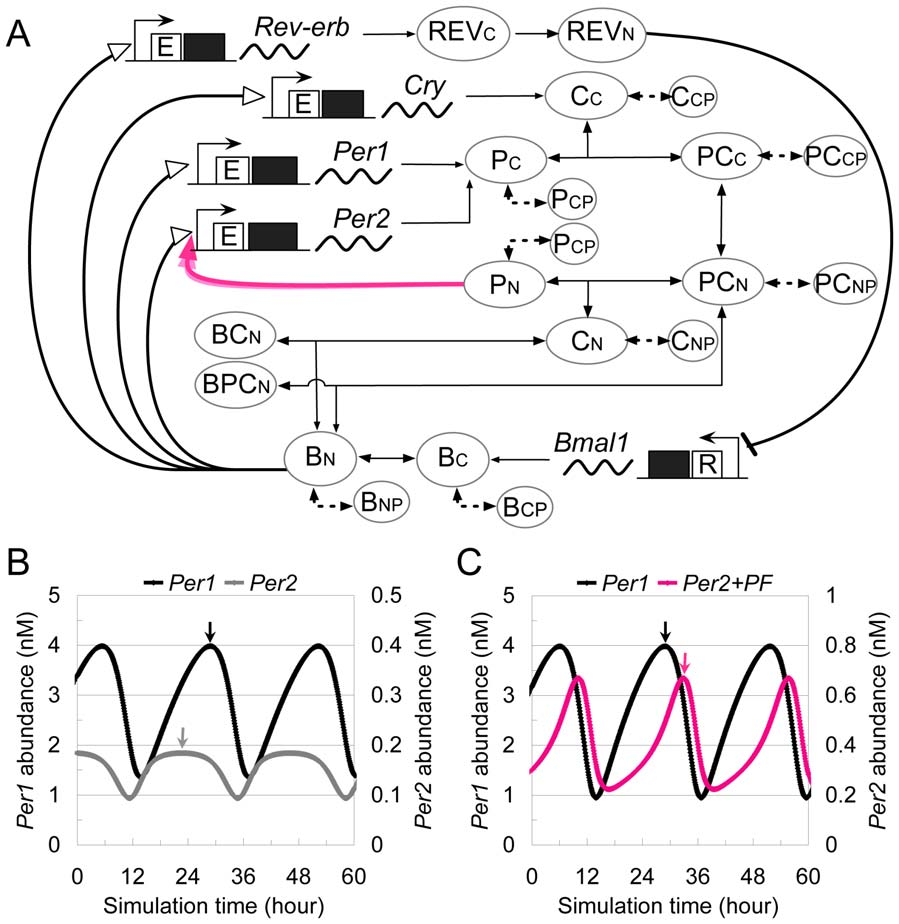
A novel model to reproduce the expression phase difference between *Per1* and *Per2*. (A) A new model hypothesized *Per2* positive feedback regulation. Nuclear PER1/2 acts as a positive regulator of *Per2* mRNA transcription. Details are described in Text S1 and parameters are indicated in Table S1. (B) A simulation result of the model without the positive feedback regulation (***k_AP2_*** = 0 h^−1^). This model simulated the circadian oscillations in 23.5-hour periods but did not reproduce the oscillation phase of *Per1* preceding that of *Per2* when applying the measured ratios of the synthesis and degradation rates of *Per1* and *Per2*. The oscillation phase of *Per1* lagged behind than that of *Per2* by 6.1 hours (6.25 hours in CT). (C) A simulation result of the model with the rate coefficient of positive feedback regulation ***k_AP2_*** = 2.4 h^−1^. This model simulated the circadian oscillations in 22.8-hour period and the expression phase of *Per2* mRNA was delayed from that of *Per1* by 4.0 hours. Arrows indicate expression peaks. After 1000 hours simulation, the first peak of *Per1* mRNA was set to simulation time 6.

To elucidate the molecular functions of nuclear PER1/2, ten reactions were additionally assumed on the basis of the model described previously: dissociation/association of the nuclear PER-CRY complex, phosphorylation/dephosphorylation/degradation of nuclear PER and CRY, and association/dissociation of the nuclear CRY with CLOCK-BMAL1 (**Figure 4A**). All kinetic parameters and reaction rate equations, including five additional variables (nuclear PER (P_N_), phosphorylated nuclear PER (P_NP_), nuclear CRY (C_N_), phosphorylated nuclear CRY (C_NP_), CRY-BMAL1 heterodimer (CB_N_)) and the modified reaction rate equation of nuclear PER-CRY complex are available in Table S1 (Model2) and Text S1.

When PER1/2 proteins (P_N_) positively regulated *Per2* transcription, the dynamics of *Per1* and *Per2* mRNA were calculated by following equations:
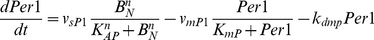
(1)

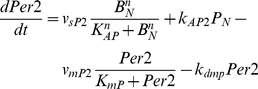
(2)where ***v_sP1_*** and ***v_sP2_*** denote the transcription rates, ***v_mP1_*** and ***v_mP2_*** are the degradation rates, ***K_mP_*** is a Michaelis-Menten coefficient, ***k_dmp_*** is a natural degradation rate, and ***k_AP2_*** is a rate coefficient of positive feedback regulation by PER1/2. The second term of Eq. 2 is the elementary form, which expresses an additional transcriptional induction of *Per2* depending on the concentration of nuclear PER1/2 proteins. The full model is governed by Eq. **1**, Eq. **2** and Eqs. S**3**–S**25** in Text S1. The model, which includes no positive feedback regulation of *Per2* by PER1/2 (i.e., ***k_AP2_*** = 0 h^−1^), reproduced 23.5-hour period oscillations corresponding to the observed period length, but it did not reproduce the phase difference between *Per1* and *Per2* with the parameters obtained experimentally (**Figure 4B**). Once a feedback induction of *Per2* transcription was introduced, the model reproduced the 4-hour phase difference (i.e., ***k_AP2_*** = 2.4 h^−1^; **Figure 4C**). This result suggested that the positive feedback regulation of *Per2* transcription by nuclear PER1/2 contributed the phase delay of *Per2 in silico*.

We also simulated the *Per1* transcriptional repression by PER1/2, which was one of the alternative ways to differentiate the promoter activity pattern of *Per1* from that of *Per2*. The oscillation phase of *Per1* expression was advanced with the increase of repression intensity; however, it did not occur ahead of *Per2* expression (**Figure S1** and Text S1; see discussion). Moreover, when the positive feedback regulation of *Per1* or the negative feedback regulation of *Per2* was assumed, the phase of *Per1* mRNA always lagged behind that of *Per2* mRNA within a range of feedback strength that can yield sustainable oscillations (see details in Text S1). In short, these three alternative models were unable to reproduce the observed phase difference between *Per1* and *Per2*.

### Positive feedback regulation by PER1/2 contributes the expression phase delay of *Per2*

**Figure 5:**
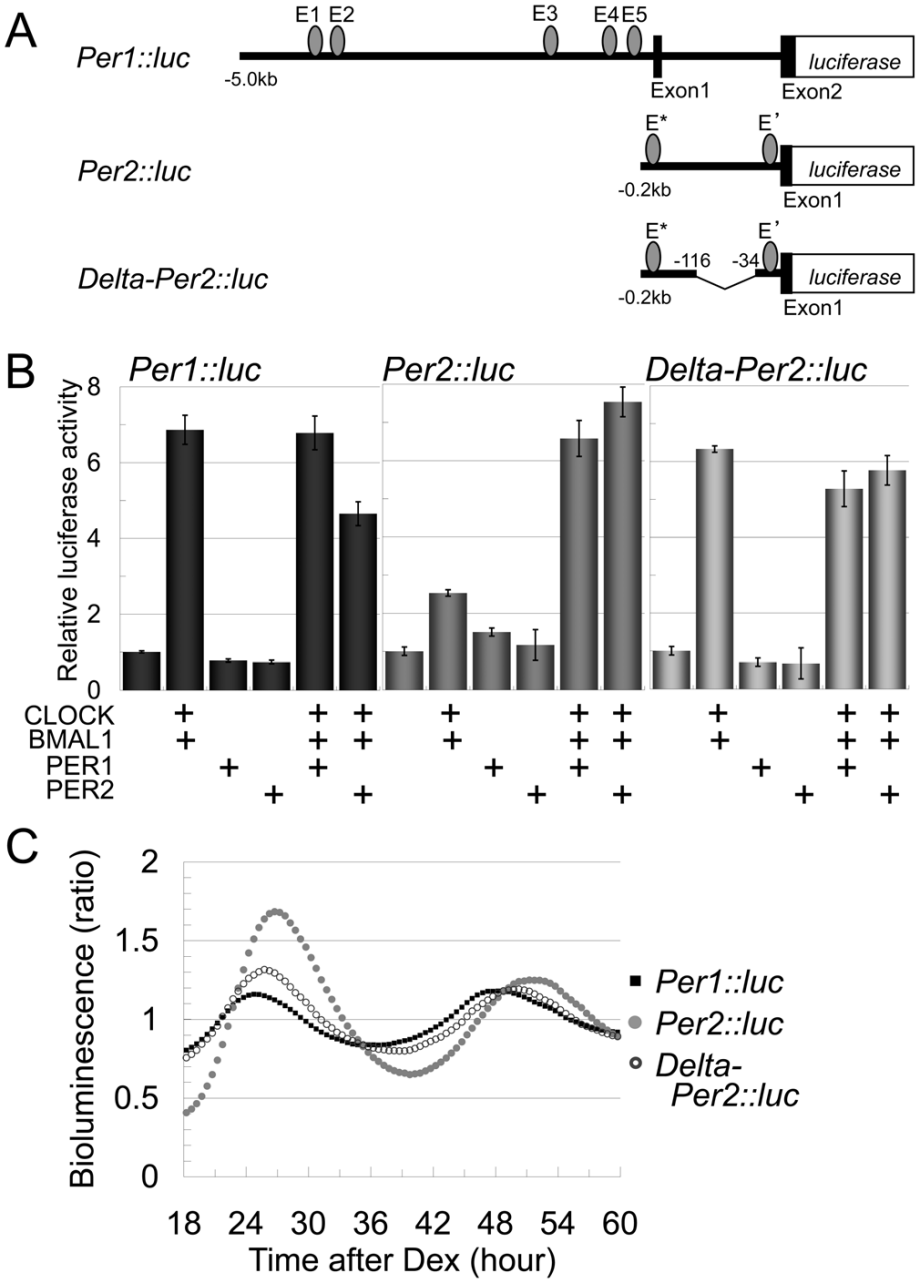
*Per2* positive feedback regulation and its contribution to oscillatory phase delay *in vitro*. (A) Schematics of *Per1::luc*, *Per2::luc* and *delta-Per2 promoter* driving luciferase reporter (*delta-Per2::luc*). *Delta-Per2::luc* does not contain the region between two E-box like elements (115-35 bp upstream from transcription start site [5] ) that contributes to positive feedback regulation. (B) PER1 and PER2 co-transfection with CLOCK and BMAL1 induced only *Per2* promoter activity. Left: *Per1::luc*, middle: *Per2::luc*, and right: *delta-Per2::luc*. Induction intensities of *Per1::luc* by CLOCK-BMAL1 were 6.86±0.38 without PER1/2, 6.78±0.44 with PER1, and 4.64±0.31 with PER2 in reference to the basal promoter activity. Induction intensities of *Per2::luc* by CLOCK-BMAL1 were 2.52±0.08 without PER1/2, 6.57±0.47 with PER1, and 7.53±0.39 with PER2 in reference to the basal promoter activity. Both PER1 and PER2 proteins significantly induced the *Per2* promoter in the presence of CLOCK-BMAL1, but not *Per1* promoter (Student's t-test, P<0.01). Induction intensities of *delta-Per2::luc* by CLOCK-BMAL1 were 6.32±0.19 without PER1/2, 5.27±0.14 with PER1, and 5.76±0.03 with PER2 in reference to the basal promoter activity, and there were no significant differences. Normalization was conducted with a pCIneo vector co-expression. Error bars indicate SEM determined from independent experiments in triplicate. (C) Representative bioluminescence oscillations of *Per1::luc* (square), *Per2::luc* (filled circle), and *deleted-Per2::luc* (open circle). The time difference from the *Per1::luc* to the *Per2::luc* expression peaks was 3.88±0.14 hours (Student's t-test, P<0.005). The phase of *delta-Per2::luc* was advanced by 2.86±0.39 hours (Student's t-test, P<0.01) compared with wild-type *Per2::luc*. Statistical data for the period and phase are described in the text and Table 1.

The positive feedback regulation by PER1/2 suggested by the simulations was examined experimentally by co-expressing the *Per1::luc* or *Per2::luc* reporters with CLOCK, BMAL1, PER1 and PER2 (**Figure 5A**). In fact, *Per1::luc* reporter activity was not affected by the presence of either PER1 or PER2, except that PER2 had a small effect on CLOCK-BMAL1 transactivation (**Figure 5B**, *left panel*). However, the co-expression of CLOCK and BMAL1 with either PER1 or PER2 resulted in an extensive induction of *Per2::luc*, while subtle inductions by PER1 and PER2 were observed (**Figure 5B**, *middle panel*). A further 3-fold increase of the CLOCK-BMAL1 transactivation of *Per2::luc* was induced by the presence of PER1 or PER2, indicating that *Per2* transcription was positively regulated by PER1/2.

To determine the significance of the positive feedback in the *Per2* oscillatory phase, we constructed a *Per2::luc* reporter that lacked the sequences required for the positive feedback regulation (*delta-Per2::luc*; **Figure 5A**). The region was located between two E-box-like elements in the *Per2* promoter and determined by Koike *et al.* (in preparation). As expected, *delta-Per2::luc* reporter activity was induced by CLOCK-BMAL1, and the induction was not intensified by either PER1 or PER2 (**Figure 5B**, *right panel*). Then, we estimated the periods and phases of bioluminescence oscillations of these reporter genes (*Per1::luc*, *Per2::luc*, *delta-Per2::luc*) when they were transfected into Rat-1 cells using a cosine fitting method (**Figure 5C**, **Table 1**). The 4-hour delay observed in *Per2::luc* compared to *Per1::luc* almost disappeared in the case of the *delta-Per2::luc* reporter in the absence of the positive feedback regulation of *Per2* transcription by PER1 and PER2 proteins. Taken together, the positive feedback regulation by PER1 and PER2 is indispensable for the phase delay of *Per2* mRNA oscillation.

**Table 1:** Oscillatory period, phase, and phase difference of promoter driving luciferase reporter.

	Period (hour)	First peak (hour)	Relative phase (CT)	Phase difference (CT)
*Per1::luc*	22.36±0.08	31.72±0.39	8.28±0.39	-
*Per2::luc*	22.99±0.04	35.65±0.16	12.16±0.14	3.88±0.14
*Delta-Per2::luc*	22.38±0.11	32.67±0.42	9.30±0.40	1.02±0.40

## Discussion

Several transcriptome analyses have revealed the circadian transcriptions of many genes with various phases [27]–[30]. The transcriptions of the mammalian clock genes *Per1* and *Per2* exhibit circadian oscillations with a phase difference of 4 hours. Jacobshagen *et al.* pointed out that extremely slow degradation of mRNA could reproduce a transcriptional phase delay [31]. In addition to the degradation rate of mRNA, our simulation analyses found that the transcription rate was also an important factor in determining the oscillatory phase. The significance of the difference in transcription was supported by the fact that the 4-hour phase difference was observed experimentally through bioluminescence oscillations of *Per1::luc* and *Per2::luc* (**Figure 5C**); the different promoters could produce the same transcriptional and translational products of the luciferase gene. Using the synthesis and degradation rates of their mRNAs, which were measured *in vitro*, we showed that the current mathematical model is not sufficient to reproduce the phase difference between *Per1* and *Per2*. Therefore, we predicted that an additional feedback regulation contributed to the phase difference.

In the model that included positive feedback regulation of *Per2*, newly synthesized PER1/2 enhanced *Per2* transcription following transactivation by CLOCK-BMAL1 and caused the delay of the transcriptional peak. More importantly, this model produced the phase lag with a slight alteration in the oscillation period, and the extent of the phase delay of *Per2* was dependent on factors that affected the intensity of positive feedback regulation, such as the abundance of PER1/2 (**Figure S2**). In addition, the circadian expressions of all genes involved in our model could be entrained to 12 h∶12 h LD cycles in which *Per1* and *Per2* transcription rate coefficients were varied in a 24-hour period square-wave manner. Significantly, the phase of *Per2* transcription also lagged behind that of *Per1* in this condition. In contrast, one of three alternative models, which included *Per1* transcriptional repression by PER1/2, could simulate the phase advance of *Per1* (**Figure S1** and Text S1), but this advance was not ahead of the *Per2* oscillation phase. The *Per2* oscillation was almost in phase with nuclear BMAL1 oscillation in the model, which implemented the synthesis and degradation rates as estimated *in vitro*, so *Per1* oscillation needed to be ahead of BMAL1 oscillation to be ahead of *Per2* oscillation. If transcriptional repression by PER1/2 surpasses CLOCK-BMAL1 transactivity at the midpoint or later within its phase, the oscillation phase of *Per1* is advanced over the peak phase of CLOCK-BMAL1. However, an increase of negative feedback strength of *Per1* transcription led to a decrease of PER1 protein expression, and our model did not simulate the expression pattern of nuclear PER1/2 that meets the requirement. Besides, the observed *Per1* mRNA oscillation is not ahead of BMAL1 protein expression peak [32]. Thus, *Per2* should be delayed to reproduce the phase difference between *Per1* and *Per2*. From these simulation results, we predicted that the positive feedback regulation of *Per2* transcription by PER1/2 could be the basis for the observed phase difference between *Per1* and *Per2*.

The hypothesis was validated by reporter analyses using *Per1::luc* and *Per2:luc*; only the *Per2* promoter, but not *Per1*, was activated by PER1/2. The significance of the positive regulation was verified further by the fact that the *Per2::luc* reporter gene that could not be transactivated by PER1/2 (*delta-Per2::luc*) lost the phase delay observed in wild-type *Per2::luc*. A recent report indicated that E*-box in *Per2* promoter contributes to 1.5-hour phase delay of *Per2* expression [33], and this might cause a residual 1-hour delay detected in *delta-Per2::luc*. However, the residual delay was not statistically significant (Student's t-test, n = 3, P>0.05). Our results strongly demonstrated that the positive feedback regulation is a major reason for the phase delay of the *Per2* mRNA oscillation.

Feedback regulation has been found in many biological systems, such as gene expression regulation and signal cascades. A recent study revealed that the positive feedback regulation slows down the kinetics of gene expression in a synthetic gene circuit and contributes to the response delay [34], indicating that the positive feedback regulation of *Per2* slows down the accumulation of PER2 protein and may affect the phase of the circadian clock. Additionally, a theoretical analysis previously demonstrated that positive feedback buffers a propagated noise without a loss of sensitivity to input signal [35]; thus, the positive feedback regulation of *Per2* could contribute to the improvement of the sensitivity to the photic signal that induces the expressions of *Per1* and *Per2* [15], [36], [37]. Although the functions of PER1 and PER2 proteins are still unclear, the positive feedback regulation of *Per2* might be involved in photoreception and the entrainment of the circadian clock.

## Materials and Methods

### Simulation experiment

The Original Leloup and Goldbeter model [22], written in Systems biology markup Language (SBML), was retrieved from BioModels Database (http://www.ebi.ac.uk/biomodels-main/BIOMD0000000074) [38]. All simulation experiments and mathematical analyses were performed in the E-Cell Simulation Environment version 3.1.106 [39]. The mathematical model consisted of simultaneous differential equations and was solved by Euler's method.

### Cell culture and measurement of mRNA half-life using real-time PCR

Total RNA was extracted from rat SCN-derived cultured cells, named RS182 [26]. A total of 1.0×10^5^ cells per 35-mm cell culture polystyrene dish (IWAKI) were proliferated in Dulbecco's modified Eagle's medium (DMEM) supplemented with 10% FBS and 1% penicillin-streptomycin at 33°C. After a 4-day proliferation period, the cells were differentiated in Neurobasal medium (Gibco) supplemented with 2% B27 supplement (Invitrogen) and 1% antibiotics (insulin-streptomycin, Invitrogen) at 39°C. Half of the dishes were treated with 10 µM actinomycin D (an mRNA synthetic inhibitor), whereas the remaining dishes were treated with DMSO (vehicle control). Total RNA was extracted at 0, 0.5, 1.0, and 2.0 hours after treatment using an RNeasy Mini Kit (Qiagen) and an RNase-Free DNase Set (Qiagen). Extracted total RNA (500 ng) was reverse-transcribed for stability with 500 µg oligo(dT)_12–18_ (Invitrogen) using SuperScript III Reverse Transcriptase (Invitrogen) according to the manufacturer's protocol. Quantification of *Per1* and *Per2* mRNAs was performed using the ABI PRISM 7900HT, SYBR Green PCR Master Mix (Applied Biosystems), and 200 nM forward/reverse primers. The primer sequences were as follows; *Per1* forward 5′- cctgg ccaat aaggc agaga -3′and reverse 5′- gcttc ttgtc tccca catgg acgat gg -3′ and *Per2* forward 5′- ggtgt ggcag ctttt gcttc -3′ and reverse 5′- cggca cagaa acgta cagtg tg -3′.

### Dual-luciferase reporter gene assay

COS-7 cells [40] were cultured in DMEM supplemented with 10% FBS, 50 mg/ml penicillin, and 50 U/ml streptomycin at 37°C. Cells were seeded the day before transfection at 4.0×10^4^ cells per well in 24-well plates and transfected with a total of 200 ng of plasmid using 1 µl of FuGENE6 (Roche). At 48 hours after transfection, cells were lysed, and luminescence was measured using the Dual-Luciferase® Reporter Assay System (Promega) and a Luminescencer-JRN II AB-2300 (ATTO BIO-INSTRUMENT) according to the manufacturer's instructions.

### Real-time monitoring of luciferase expression in cultured cells

Rat-1 cells were cultured in DMEM supplemented with 10% FBS and penicillin-streptomycin at 37°C. Cells were seeded 48 hours before transfection at 4.0×10^5^ cells per dish with 2 ml of medium in 35-mm dishes and transfected with 1.6 µg of plasmid using 8 µl of FuGENE6 (Roche). After 24 hours, the medium was replaced with culture medium supplemented with 100 µM luciferin. At 45 hours after transfection, cells were treated with 100 nM dexamethasone for 3 hours, and then the medium was replaced with culture medium containing 100 µM luciferin. Bioluminescence was measured with photomultiplier tube detector assemblies (LM2420; Hamamatsu). The time series bioluminescent data of triplicate samples, which were measured from 0.5 to 3.8 days after the medium change, were fitted to a cosine curve using R version 2.9.1.

## Supporting Information

Figure S1 **Analysis of the effect of PER1/2 negative feedback regulation on expression period and phase.** The *Per1* mRNA expression phase variation that depended on the intensity of additional PER1/2 negative feedback regulation, was mathematically simulated using the negative feedback regulation model (see Text S1). (A) Schematic representation of a model hypothesized *Per1* negative feedback regulation. (B) The oscillation period of *Per1* was increased by 12 hours, while the phase difference between *Per1* and *Per2* varied by 6 hours. (C) The *Per1* expression phase advanced as the negative feedback strength became larger. However, the phase advance was saturated when the expression phase of *Per1* was close to that of *Per2*. X-axis: strength of the negative feedback regulation, namely the rate coefficient, ***k_RP1_***, of the transcriptional equation (Text S1, Eq. **S1b**, the first term).
(TIF)

Figure S2 **Analysis of the effect of PER positive feedback regulation on expression period and phase.** The *Per2* mRNA expression phase variation that depended on the intensity of additional PER1/2 positive feedback regulation was mathematically simulated using the positive feedback regulation model (see Text S1). X-axis: strength of the positive feedback regulation, namely the rate coefficient, ***k_AP2_***, of the transcriptional equation (Text S1, Eq. **S2a**, the second term). (A) The oscillation period of *Per2* varied within ±1 hour, while the phase difference between *Per1* and *Per2* varied ±6 hours. (B) The *Per2* expression phase lagged behind the *Per1* expression phase when the strength of positive feedback regulation caused ***k_AP2_*** to be greater than or equal to 0.8 h^−1^, and stronger positive feedback regulation increased the phase difference.
(TIF)

Text S1 (DOC)

Table S1 (DOC)
