# Supplementary figures and images for: Positive Autoregulation Delays the Expression Phase of Mammalian Clock Gene Per2
Source: PLoS One. 2011 Apr 14;6(4):e18663. doi: 10.1371/journal.pone.0018663 (PMC3077398; doi:10.1371/journal.pone.0018663)

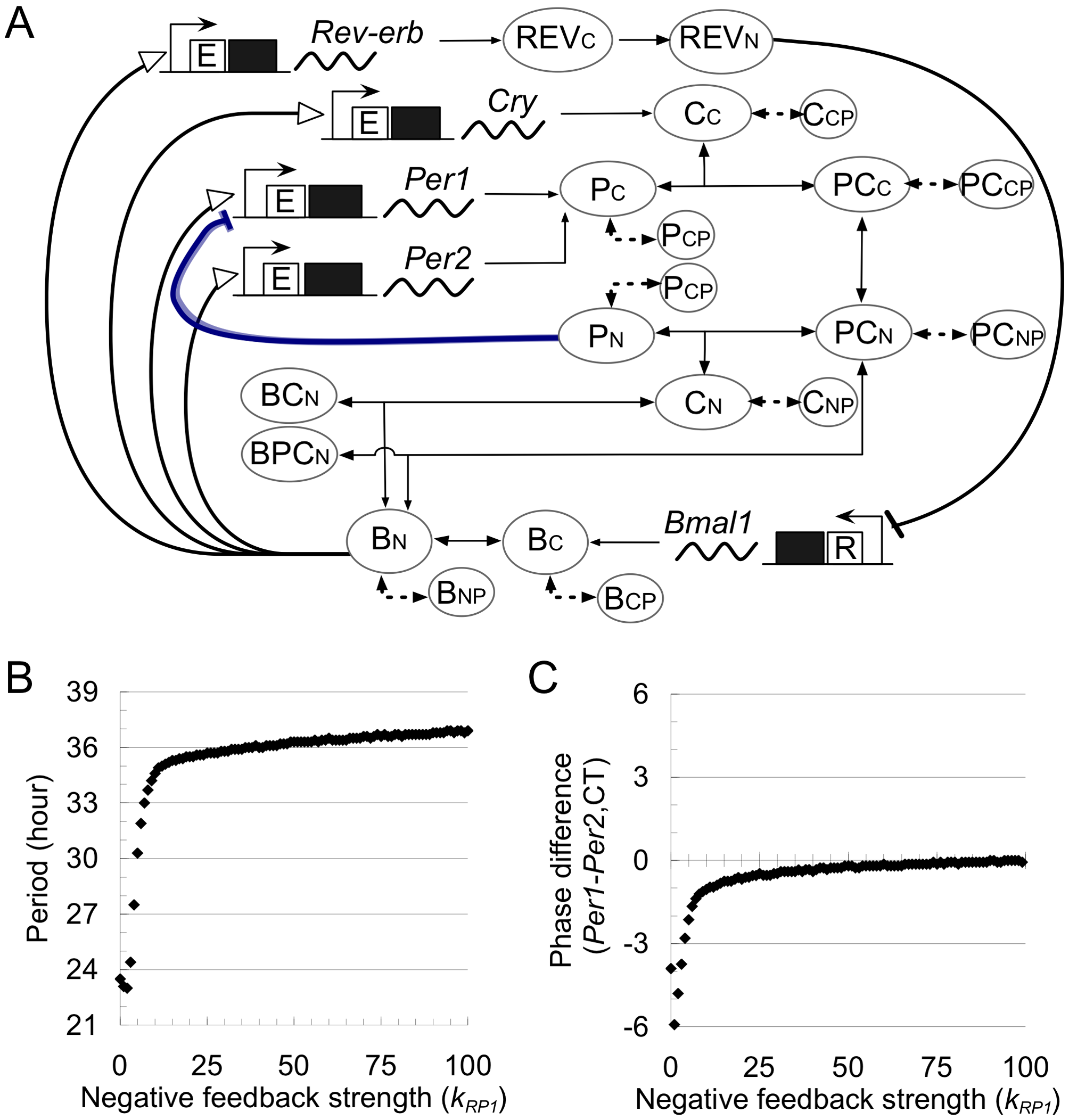

Supplement: Figure S1 — Analysis of the effect of PER1/2 negative feedback regulation on expression period and phase. The Per1 mRNA expression phase variation that depended on the intensity of additional PER1/2 negative feedback regulation, was mathematically simulated using the negative feedback regulation model (see Text S1). (A) Schematic representation of a model hypothesized Per1 negative feedback regulation. (B) The oscillation period of Per1 was increased by 12 hours, while the phase difference between Per1 and Per2 varied by 6 hours. (C) The Per1 expression phase advanced as the negative feedback strength became larger. However, the phase advance was saturated when the expression phase of Per1 was close to that of Per2. X-axis: strength of the negative feedback regulation, namely the rate coefficient, kRP1, of the transcriptional equation (Text S1, Eq. S1b, the first term). (TIF) [file pone.0018663.s001.tif]

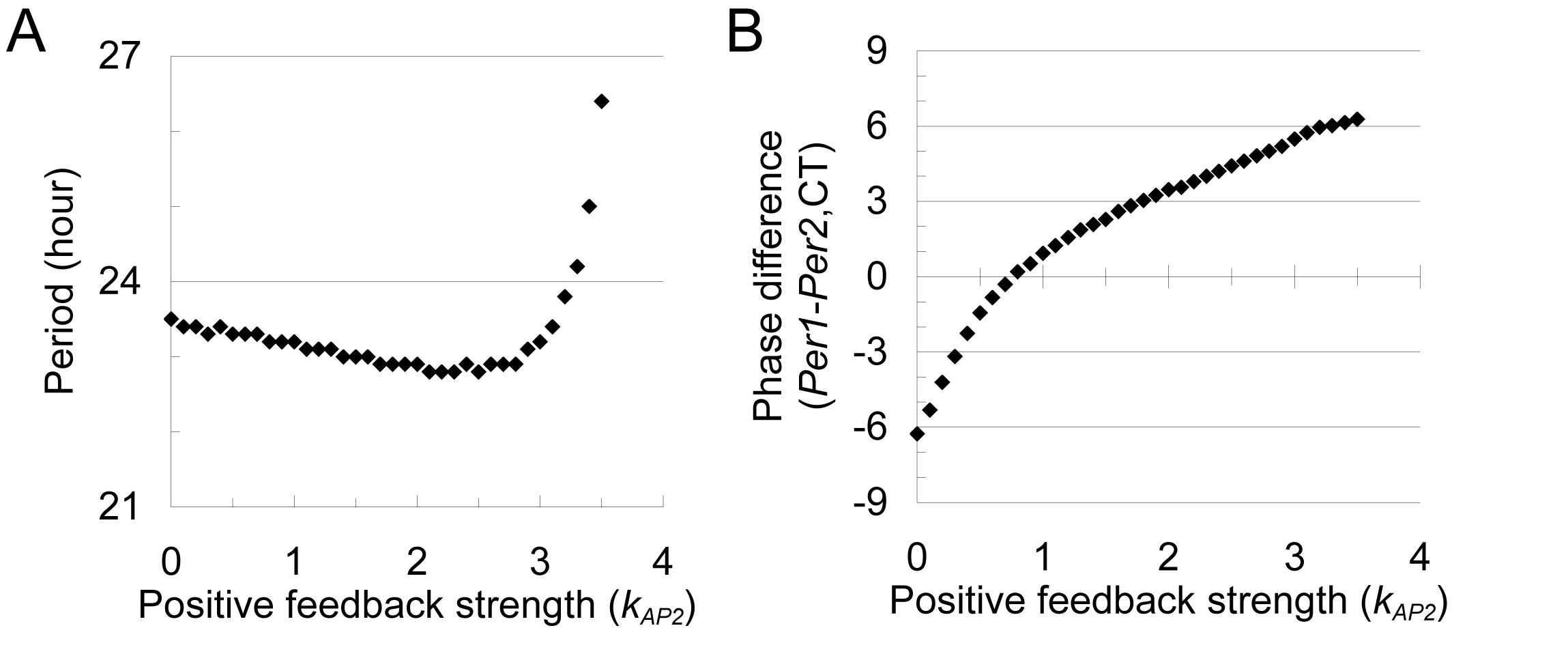

Supplement: Figure S2 — Analysis of the effect of PER positive feedback regulation on expression period and phase. The Per2 mRNA expression phase variation that depended on the intensity of additional PER1/2 positive feedback regulation was mathematically simulated using the positive feedback regulation model (see Text S1). X-axis: strength of the positive feedback regulation, namely the rate coefficient, kAP2, of the transcriptional equation (Text S1, Eq. S2a, the second term). (A) The oscillation period of Per2 varied within ±1 hour, while the phase difference between Per1 and Per2 varied ±6 hours. (B) The Per2 expression phase lagged behind the Per1 expression phase when the strength of positive feedback regulation caused kAP2 to be greater than or equal to 0.8 h−1, and stronger positive feedback regulation increased the phase difference. (TIF) [file pone.0018663.s002.tif]
