# Supplementary material for: Positive Autoregulation Delays the Expression Phase of Mammalian Clock Gene Per2
Source: PLoS One. 2011 Apr 14;6(4):e18663. doi: 10.1371/journal.pone.0018663 (PMC3077398; doi:10.1371/journal.pone.0018663)
Supplement: Text S1 — (DOC) [file pone.0018663.s003.doc]

**Supporting Information**

**A mathematical model of the mammalian circadian oscillatory network for comparing the expression phases of two *Per* genes (Model1)*.***

The mRNA expression phases of *Period1* (*Per1*) and *Period2* (*Per2*) are calculated using the mathematical model schematized in Figure 1A, which is based on the model proposed by Leloup and Goldbeter in 2003 [1]. The original model incorporates the following cellular processes: transcription, translation, and degradation of *Period* (*Per*), *Cryptochrome* (*Cry*), *Bmal1* and *Rev-erbα* mRNAs; reversible formation of unphosphorylated PER-CRY complex and PER-CRY-BMAL1 complex; reversible phosphorylation of PER, CRY, PER-CRY and BMAL1; and reversible nuclear entry of unphosphorylated PER-CRY, BMAL1 and REV-ERB*α*. PER, CRY, REV-ERB*α*, BMAL1, PER-CRY complex, and BMAL1-PER-CRY complex are denoted by P, C, R, B, PC, and BPC, respectively, in schematics and equations. In addition, the subscript letters C, N, CP, and NP denote cytoplasmic protein, nuclear protein, phosphorylated cytoplasmic protein, and phosphorylated nuclear protein, respectively. For simplicity, the *Cry1* and *Cry2* genes are represented by the single *Cry* gene. Because CLOCK protein is constitutively expressed at a high level, BMAL1 protein is assumed to form a complex with CLOCK immediately and maintain this complex. The PER1 and PER2 proteins are represented by the single PER protein. The cellular abundances of *Per1* and *Per2* mRNAs are calculated by the same differential equation as for the original *Per* mRNA. All kinetics parameters are available in Table S1 (Model1), and all kinetic equations for the 20 variables are described below:

(S1)

(S2)

(S3)

(S4)

(S5)

(S6)

(S7)

(S8)

(S9)

(S10)

(S11)

(S12)

(S13)

(S14)

(S15)

(S16)

(S17)

(S18)

(S19)

(S20)

**A modified mathematical model introduced a nuclear PER and CRY monomer to simulate the expression phase difference between *Per1* and *Per2* mRNA expressions (Model2).**

To introduce an additional mechanism of transcriptional regulation by a nuclear PER monomer, several assumptions were introduced to the model described above. The dissociation of the nuclear PER-CRY complex (***PC_N_***) was described in the same equation, and parameters such as cytoplasmic PER-CRY (***PC_C_***) and nuclear PER (***P_N_***) and CRY (***C_N_***) were degraded in the same manner as the nuclear PER-CRY complex instead of by the PER-CRY degradation processes. Nuclear CRY associates and dissociates with nuclear CLOCK-BMAL1 (***B_N_***) in the same manner as nuclear PER-CRY. Changes in five additionally introduced molecules (nuclear PER (***P_N_***), nuclear CRY (***C_N_***), nuclear phosphorylated PER (***P_NP_***), nuclear phosphorylated CRY (***C_NP_***), and BMAL1-CRY complex (***BC_N_***)) were calculated by the following equations:

(S21)

(S22)

(S23)

(S24)

(S25)

Accordingly, the kinetic equation of nuclear PER-CRY complex (***PC_N_***, Eq. **S11**) and nuclear BMAL1 (***B_N_***, Eq. **S17**) were replaced by Eq. **S11’** and Eq. **S17'**, respectively:

(S11’)

(S17’)

All kinetics parameters are available in Table S1 (Model2).

**The *Per2* positive feedback regulation model.**

This model is based on the modified model described above (Eqs. **S1-S25**) and tests the hypothesis that nuclear PER acts as a positive regulator of *Per2* mRNA transcription. The positive feedback regulation of *Per2* transcription by nuclear PER (***P_N_***) is described in a mass action law. The *Per2* mRNA kinetic equation (Eq. **S2**) is replaced by Eq. **S2a**:

(S2a)

where ***k_AP2_*** denotes a rate coefficient of positive feedback regulation of *Per2* transcription by PER. In this model including the positive feedback regulation of *Per2* transcription by PER proteins (***k_AP2_*** > 0), bistability appeared as the fluctuating orbits kept stable oscillation or converged to a stable steady state depending on the initial values of variables.

**The *Per1* positive feedback regulation model.**

This model tests the hypothesis that nuclear PER2 acts as a positive regulator of *Per1* mRNA transcription. The positive feedback regulation of *Per1* transcription by nuclear PER (***P_N_***) is described in a mass action law. The *Per1* mRNA kinetic equations (Eq. **S1**) is replaced by Eq. **S1a**:

(S1a)

where ***k_AP1_*** denotes a rate coefficient of positive feedback regulation of *Per1* transcription by PER. The circadian oscillations are simulated when ***k_AP1_*** is a value from 0.01 to 0.92 h^-1^. The full model is governed by Eq. **S1a** and Eqs. S**2**-S**25***.*

**The *Per2* negative feedback regulation model.**

This model tests the hypothesis that nuclear PER acts as a negative regulator of *Per2* mRNA transcription, the negative feedback regulation of *Per2* transcription by nuclear PER (***P_N_***) is introduced into the original transcriptional term. The *Per2* mRNA kinetic equation (Eq. S**2**) is replaced by Eq. **S2b**:

(S2b)

where ***k_RP2_*** denotes the strength of negative feedback regulation of *Per2* transcription by PER. ***k_RP2_*** can be varied from 0 to more than 10000. The full model is governed by Eq. **S1**, Eq. **S2b** and Eqs. S**3**-S**25***.*

**The *Per1* negative feedback regulation model.**

This model is governed by tests the hypothesis that nuclear PER acts as a negative regulator of *Per1* mRNA transcription. The negative feedback regulation of *Per1* transcription by nuclear PER (***P_N_***) is introduced into the original transcriptional term. The *Per1* mRNA kinetic equation (Eq. **S1**) is replaced by Eq. **S1b**:

(S1b)

where ***k_RP1_*** denotes the strength of negative feedback regulation of *Per1* transcription by PER. ***k_RP1_*** can be varied from 0 to more than 10000. The full model is governed by Eq. **S1b**and Eqs. S**2**-S**25***.*

1. Leloup JC, Goldbeter A (2003) Toward a detailed computational model for the mammalian circadian clock. Proc Natl Acad Sci USA 100: 7051-7056.
