# Supplementary material for: Positive Autoregulation Delays the Expression Phase of Mammalian Clock Gene Per2
Source: PLoS One. 2011 Apr 14;6(4):e18663. doi: 10.1371/journal.pone.0018663 (PMC3077398; doi:10.1371/journal.pone.0018663)
Supplement: Table S1 — (DOC) [file pone.0018663.s004.doc]

**Table S1. The parameter set for simulation of the circadian regulatory network model.**

| **Parameter** | **Model1** | **Model2** | **Unit** | **Description** |
| --- | --- | --- | --- | --- |
| ***v_sP1_*** | **2.4** | **2.4** | nM˙h^-1^ | Transcription rate coefficient of *Per1* |
| ***v_sP2_*** | **2.4** | **0.8** | nM˙h^-1^ | Transcription rate coefficient of *Per2* |
| ***v_sC_*** | **2.2** | **2.2** | nM˙h^-1^ | Transcription rate coefficient of *Cry* |
| ***v_sR_*** | **1.6** | **1.6** | nM˙h^-1^ | Transcription rate coefficient of *Rev-erbα* |
| ***v_sB_*** | **1.8** | **1.8** | nM˙h^-1^ | Transcription rate coefficient of *Bmal1* |
| ***K_AP_*** | **0.6** | **0.6** | nM | Transcription equilibrium constant of *Per1*, *Per2* |
| ***K_AC_*** | **0.6** | **0.6** | nM | Transcription equilibrium constant of *Cry* |
| ***K_AR_*** | **0.6** | **0.6** | nM | Transcription equilibrium constant of *Rev-erbα* |
| ***K_IB_*** | **1.0** | **1.0** | nM | Transcription equilibrium constant of *Bmal1* |
| ***n*** | **2** | **2** | **-** | Hill coefficient of *Per1*, *Per2* and *Cry* transcription |
| ***h*** | **2** | **2** | **-** | Hill coefficient of *Rev-erbα* transcription |
| ***m*** | **2** | **2** | **-** | Hill coefficient of *Bmal1* transcription |
| ***v_mP1_*** | **2.2** | **2.2** | nM˙h^-1^ | mRNA degradation rate coefficient of *Per1* |
| ***v_mP2_*** | **2.2** | **1.98** | nM˙h^-1^ | mRNA degradation rate coefficient of *Per2* |
| ***v_mC_*** | **2.0** | **2.0** | nM˙h^-1^ | mRNA degradation rate coefficient of *Cry* |
| ***v_mR_*** | **1.6** | **1.6** | nM˙h^-1^ | mRNA degradation rate coefficient of *Rev-erbα* |
| ***v_mB_*** | **1.3** | **1.3** | nM˙h^-1^ | mRNA degradation rate coefficient of *Bmal1* |
| ***K_mP_*** | **0.3** | **0.3** | nM˙h^-1^ | Michaelis-Menten coefficient of mRNA degradation of *Per1, Per2* |
| ***K_mC_*** | **0.4** | **0.4** | nM | Michaelis-Menten coefficient of mRNA degradation of *Cry* |
| ***K_mR_*** | **0.4** | **0.4** | nM | Michaelis-Menten coefficient of mRNA degradation of *Rev-erbα* |
| ***K_mB_*** | **0.4** | **0.4** | nM | Michaelis-Menten coefficient of mRNA degradation of *Bmal1* |
| ***k_dmp_*** | **0.02** | **0.02** | h^-1^ | Rate coefficient of mRNA natural degradation of *Per1, Per2* |
| ***k_dmc_*** | **0.02** | **0.02** | h^-1^ | Rate coefficient of *Cry* mRNA natural degradation |
| ***k_dmr_*** | **0.02** | **0.02** | h^-1^ | Rate coefficient of *Rev-erbα* mRNA natural degradation |
| ***k_dmb_*** | **0.02** | **0.02** | h^-1^ | Rate coefficient of *Bmal1* mRNA natural degradation |
| ***k_sP_*** | **0.6** | **1.2** | h^-1^ | Translation rate coefficient of *Per1*, *Per2* |
| ***k_sC_*** | **3.2** | **3.2** | h^-1^ | Translation rate coefficient of *Cry* |
| ***k_sR_*** | **1.7** | **1.7** | h^-1^ | Translation rate coefficient of *Rev-erbα* |
| ***k_sB_*** | **0.32** | **0.32** | h^-1^ | Translation rate coefficient of *Bmal1* |
| ***V_1P_*** | **9.6** | **9.6** | nM˙h^-1^ | Phosphorylation rate coefficient of cytoplasmic PER |
| ***V_2P_*** | **0.6** | **0.6** | nM˙h^-1^ | Dephosphorylation rate coefficient of cytoplasmic PER |
| ***V_3P_*** | **-** | **2.4** | nM˙h^-1^ | Phosphorylation rate coefficient of nuclear PER |
| ***V_4P_*** | **-** | **0.2** | nM˙h^-1^ | Dephosphorylation rate coefficient of nuclear PER |
| ***V_1C_*** | **1.2** | **1.2** | nM˙h^-1^ | Phosphorylation rate coefficient of cytoplasmic CRY |
| ***V_2C_*** | **0.2** | **0.2** | nM˙h^-1^ | Dephosphorylation rate coefficient of cytoplasmic CRY |
| ***V_3C_*** | **-** | **1.2** | nM˙h^-1^ | Phosphorylation rate coefficient of nuclear CRY |
| ***V_4C_*** | **-** | **0.2** | nM˙h^-1^ | Dephosphorylation rate coefficient of nuclear CRY |
| ***V_1PC_*** | **2.4** | **2.4** | nM˙h^-1^ | Phosphorylation rate coefficient of cytoplasmic PER-CRY |
| ***V_2PC_*** | **0.2** | **0.2** | nM˙h^-1^ | Dephosphorylation rate coefficient of cytoplasmic PER-CRY |
| ***V_3PC_*** | **2.4** | **2.4** | nM˙h^-1^ | Phosphorylation rate coefficient of nuclear PER-CRY |
| ***V_4PC_*** | **0.2** | **0.2** | nM˙h^-1^ | Dephosphorylation rate coefficient of nuclear PER-CRY |
| ***V_1B_*** | **1.4** | **1.4** | nM˙h^-1^ | Phosphorylation rate coefficient of cytoplasmic BMAL1 |
| ***V_2B_*** | **0.2** | **0.2** | nM˙h^-1^ | Dephosphorylation rate coefficient of cytoplasmic BMAL1 |
| ***V_3B_*** | **1.4** | **1.4** | nM˙h^-1^ | Phosphorylation rate coefficient of nuclear BMAL1 |
| ***V_4B_*** | **0.4** | **0.4** | nM˙h^-1^ | Dephosphorylation rate coefficient of nuclear BMAL1 |
| ***K_p_*** | **1.006** | **1.006** | nM | Michaelis-Menten coefficient of phosphorylation |
| ***K_dp_*** | **0.1** | **0.1** | nM | Michaelis-Menten coefficient of dephosphorylation |
| ***k_1_*** | **0.8** | **0.8** | h^-1^ | Nuclear import rate coefficient of PER-CRY |
| ***k_2_*** | **0.4** | **0.4** | h^-1^ | Nuclear export rate coefficient of PER-CRY |
| ***k_3_*** | **0.8** | **0.8** | nM^-1^˙h^-1^ | Association reaction rate coefficient of PER-CRY |
| ***k_4_*** | **0.4** | **0.4** | h^-1^ | Dissociation reaction rate coefficient of PER-CRY |
| ***k_5_*** | **0.8** | **0.8** | nM^-1^˙h^-1^ | Nuclear import rate coefficient of BMAL1 |
| ***k_6_*** | **0.4** | **0.4** | h^-1^ | Nuclear export rate coefficient of BMAL1 |
| ***k_7_*** | **1.0** | **1.0** | nM^-1^˙h^-1^ | Association reaction rate coefficient of nuclear PER-CRY and BMAL1 |
| ***k_8_*** | **0.2** | **0.2** | h^-1^ | Dissociation reaction rate coefficient of nuclear PER-CRY and BMAL1 |
| ***k_9_*** | **1.0** | **1.0** | nM^-1^˙h^-1^ | Nuclear import rate coefficient of REV-ERBα |
| ***k_10_*** | **0.2** | **0.2** | h^-1^ | Nuclear export rate coefficient of REV-ERBα |
| ***k_11_*** | **-** | **0.8** | nM^-1^˙h^-1^ | Association reaction rate coefficient of nuclear PER-CRY |
| ***k_12_*** | **-** | **0.4** | h^-1^ | Dissociation reaction rate coefficient of nuclear PER-CRY |
| ***k_13_*** | **-** | **1.0** | nM^-1^˙h^-1^ | Association reaction rate coefficient of nuclear CRY and BMAL1 |
| ***k_14_*** | **-** | **0.2** | h^-1^ | Dissociation reaction rate coefficient of nuclear CRY and BMAL1 |
| ***v_dPC_*** | **3.4** | **3.4** | nM˙h^-1^ | Degradation rate coefficient of PER |
| ***v_dCC_*** | **1.4** | **1.4** | nM˙h^-1^ | Degradation rate coefficient of CRY |
| ***v_dPN_*** | **-** | **1.4** | nM˙h^-1^ | Degradation rate coefficient of nuclear PER |
| ***v_dCN_*** | **-** | **1.4** | nM˙h^-1^ | Degradation rate coefficient of nuclear CRY |
| ***v_dPCC_*** | **1.4** | **1.4** | nM˙h^-1^ | Degradation rate coefficient of cytoplasmic PER-CRY |
| ***v_dPCN_*** | **1.4** | **1.4** | nM˙h^-1^ | Degradation rate coefficient of nuclear PER-CRY |
| ***v_dRC_*** | **4.4** | **4.4** | nM˙h^-1^ | Degradation rate coefficient of cytoplasmic REV-ERBα |
| ***v_dRN_*** | **0.8** | **0.8** | nM˙h^-1^ | Degradation rate coefficient of nuclear REV-ERBα |
| ***v_dBC_*** | **3.0** | **3.0** | nM˙h^-1^ | Degradation rate coefficient of cytoplasmic BMAL1 |
| ***v_dBN_*** | **3.0** | **3.0** | nM˙h^-1^ | Degradation rate coefficient of nuclear BMAL1 |
| ***v_dI_*** | **1.6** | **1.6** | nM˙h^-1^ | Degradation rate coefficient of PER-CRY-BMAL1 |
| ***v_dBCN_*** | **-** | **1.6** | nM˙h^-1^ | Degradation rate coefficient of CRY-BMAL1 |
| ***K_d_*** | **0.3** | **0.3** | nM | Michaelis-Menten coefficient of protein degradation |
| ***k_dn_*** | **0.02** | **0.02** | h^-1^ | Rate coefficient of protein natural degradation |
| ***k_AP2_*** | **-** | **2.4** | h^-1^ | Reaction rate coefficient of positive feedback regulation of *Per2* |
